# Dietary Antioxidant Supplements and Uric Acid in Chronic Kidney Disease: A Review

**DOI:** 10.3390/nu11081911

**Published:** 2019-08-15

**Authors:** Stefanos Roumeliotis, Athanasios Roumeliotis, Evangelia Dounousi, Theodoros Eleftheriadis, Vassilios Liakopoulos

**Affiliations:** 1 Division of Nephrology and Hypertension, 1st Department of Internal Medicine, AHEPA Hospital, School of Medicine, Aristotle University of Thessaloniki, Thessaloniki 54636, Greece; 2 Department of Nephrology, School of Medicine, University of Ioannina, Ioannina 45110, Greece; 3 Department of Nephrology, School of Medicine, University of Thessaly, Larissa 41110, Greece

**Keywords:** antioxidants, chronic kidney disease, hyperuricemia, oxidative stress, uric acid

## Abstract

Increased serum levels of uric acid have been associated with the onset and development of chronic kidney disease (CKD), cardiovascular disease, and mortality, through several molecular pathogenetic mechanisms, such as inflammation and oxidative stress. Oxidative stress is present even in the early stages of CKD, progresses parallelly with the deterioration of kidney function, and is even more exacerbated in end-stage renal disease patients undergoing maintenance hemodialysis. Although acting in the plasma as an antioxidant, once uric acid enters the intracellular environment; it behaves as a powerful pro-oxidant. Exogenous intake of antioxidants has been repeatedly shown to prevent inflammation, atherosclerosis and oxidative stress in CKD patients. Moreover, certain antioxidants have been proposed to exert uric acid-lowering properties. This review aims to present the available data regarding the effects of antioxidant supplements on both oxidative stress and uric acid serum levels, in a population particularly susceptible to oxidative damage such as CKD patients.

## 1. Introduction

In humans, uric acid is the final product of purine catabolism, excreted mainly by the kidneys through tubular transporters. The enzyme xanthine oxidase (XO) is responsible for generating uric acid by catalyzing the oxidation process of hypoxanthine to xanthine and then to uric acid [1]. Due to its renal excretion, any decrease in the glomerular filtration rate is accompanied by subsequent retention of uric acid. To counteract the impaired renal function in chronic kidney disease (CKD), there is a compensatory gut uric acid removal, though not adequate as CKD progresses. As a result, serum uric acid increases, with approximately 50% of patients starting hemodialysis (HD) being hyperuricemic [2]. Accumulating evidence suggests that hyperuricemia is not only a result but also a risk factor for CKD [3], through several molecular pathogenetic mechanisms. Besides impaired renal function, other conditions associated with cardiovascular disease (CVD) and high mortality, like obesity, diabetes, hypertension, and metabolic syndrome, have emerged as risk factors for hyperuricemia.

The mechanisms underlying the association between hyperuricemia and mortality, CVD, renal injury, and progression of CKD are yet unclear. Recently a close relationship between uric acid and oxidative stress (OS) has been highlighted. OS is defined as the imbalance between antioxidants and the production of pro-oxidants in favor of the latter, with subsequent injury in tissues and organs. Accumulation of oxidants such as reactive oxygen species (ROS) leads to oxidation of DNA, proteins, carbohydrates, and lipids, cell apoptosis, and organ dysfunction. To counteract the hazardous effects of oxidants, the human body excretes antioxidants that bind directly to pro-oxidants, which neutralizes them and prevents any damage. OS is present even in the early stages of CKD, progresses parallelly with the deterioration of kidney function, and is even more exacerbated in end-stage renal disease (ESRD) patients undergoing maintenance HD [4]. Moreover, it is also recognized as a novel risk factor of mortality and CVD in this population. Therefore, the interrelationship between uric acid, OS, and endothelial injury—especially in CKD patients—is of great importance.

Exogenous intake of antioxidants has been repeatedly shown to prevent inflammation, atherosclerosis, and OS in CKD and dialysis patients [5,6]. Moreover, certain antioxidants have been proposed to exert uric acid-lowering properties. This review aims to present the available data regarding the effects of antioxidant supplements on OS and uric acid serum levels, especially in a population particularly susceptible to oxidative damage such as CKD patients.

## 2. Uric Acid and CKD

There is a growing body of associative evidence suggesting causation between hyperuricemia and progression of renal disease, through several pathogenetic mechanisms. Firstly, the precipitation of uric crystals in the glomeruli can cause direct renal damage. However, it has been shown that increased uric acid in serum can cause glomerular hypertrophy and systemic/glomerular hypertension through a crystal-independent mechanism [7]. Furthermore, several researchers suggest that the deterioration of renal function observed in hyperuricemic patients might be due to co-existing conditions such as vascular calcification, obesity and hypertension and not to the elevated serum uric acid per se [8]. Other mechanisms include uric acid-derived hypertension, endothelial dysfunction, inflammation and both renal and systemic OS.

In vitro, uric acid triggered a phenotypic alteration of renal tubular cells with subsequent deterioration of renal function and production of intrarenal inflammation and OS markers [9]. In rats, chemically-induced hyperuricemia was associated with decreased renal function mainly due to enhanced OS, systemic and glomerular hypertension, glomerular hypertrophy, and vascular damage [10,11]. The deleterious effect of hypertension on oxidative balance and the kidney is well-established [12]. Similarly, Johnson et al. suggested that even in the case of mild hypertension, elevated serum levels of uric acid might lead to systemic and renal endothelial dysfunction, loss of kidney autoregulation, and subsequently the development of albuminuria and progressive decrease in estimated glomerular filtration rate (eGFR) [13]. The effects of uric acid in the kidney are summarized in Table 1.

Data from animal models suggest that hyperuricemia might trigger the progression of CKD mainly by causing glomerular and systemic hypertension. However, in CKD, this pathogenetic pathway is secondary, since the rise of blood pressure is primarily due to water and sodium retention. That might be the reason why, in patients with renal disease, uric acid was not found to be an independent predictor of disease progression [18,19]. On the other hand, data from large epidemiological studies show that even in subjects with normal kidney function, hyperuricemia is a strong risk factor for the onset of CKD [3].

In various populations with renal dysfunction, uric acid has been associated with adverse renal outcomes. In type 1 and 2 diabetics, high uric acid was repeatedly shown to be a strong and independent predictor of diabetic kidney disease progression [20,21,22]. Data from prospective and cross-sectional studies suggest a clear, dose-responsive association between uric acid and the risk of progression of diabetic nephropathy, defined as a decline in eGFR and development of albuminuria [23,24].

In IgA nephropathy patients, serum uric acid has been repeatedly shown to be closely associated with renal prognosis. Several investigators highlighted that serum uric acid was a novel, previously underestimated risk factor for deterioration of renal function and adverse outcomes in patients with IgA nephropathy [25,26,27], predicting renal injuries associated with enhanced OS such as tubulointerstitial injury and diffuse proliferative glomerulonephritis [28]. 

Akalin et al. followed 307 patients who had received a kidney transplant for 4.3 years. The authors found that after adjustment for several risk factors including age, sex, race, and receiving a cadaveric kidney, new onset of CKD—defined as eGFR < 50 mL/min—was strongly associated with high serum uric acid levels. Moreover, at the end of follow-up, hyperuricemia was a strong and independent predictor of cardiovascular events, graft failure, and chronic allograft nephropathy [29].

To investigate whether elevated serum uric acid is associated with onset of CKD, Li et al. conducted a systematic review and meta-analysis, including 13 studies and 190,718 subjects. After long-term (defined as over five years) follow-up of subjects without CKD, high serum uric acid levels were found to be a strong, independent risk factor for the development of new-onset CKD with an odds ratio of 2.35 (95% CI, 1.59–3.46), [30]. Moreover, a large population-based retrospective cohort study in 111,992 hyperuricemic patients showed that after three years of follow-up, those who achieved serum uric acid levels below 6 mg/dL exhibited a 37% reduction in renal events (defined as a reduction in eGFR more than 30 mL/min or onset of ESRD) [31]. Su et al. performed another systematic review and meta-analysis to explore the effects of treatment with urate-lowering therapy on the major clinical outcomes of kidney disease. Sixteen prospective, randomized controlled studies were included with 1211 CKD patients. The authors found that treatment with uric-acid lowering agents was accompanied by a significant 60% reduction in relative risk (RR) for cardiovascular events (95% CI, 17–62) and a 55% RR reduction in kidney failure related events (95% CI, 31–64) [32].

## 3. Uric Acid and OS

Hyperuricemia is a risk factor for the onset and development of CKD and adverse outcome in CKD patients. One of the most crucial effects of uric acid in the kidney is the triggering of OS. 

Uric acid acts as a strong antioxidant in plasma, but after entering the cell environment, it promotes OS. In vitro and in vivo studies have shown that uric acid is one of the most selective antioxidants in plasma, capable of neutralizing important and dangerous pro-oxidants such as peroxynitrite, hydroxyl, and iron-containing free radicals. Furthermore, it is thought to be the most important antioxidant in plasma, conferring about 60% to the total plasma antioxidant capacity in humans [33]. At physiological levels, uric acid abrogates the organ injury mediated by activated polymorphonuclear blood and erythrocyte cells, that are well-established producers of free radicals [34]. Superoxide dismutase (SOD) is an effective antioxidant that prevents damage from free reactive oxygen species. Data from in vitro and in vivo studies suggest that uric acid preserves the structure and function of extracellular SOD, by preventing its oxidative neutralization mediated by atherosclerosis [35,36]. Another antioxidant function of uric acid is that it protects DNA from oxidative damage and mediates the repair of any nuclear area that was exposed to oxidation [37]. It has also been suggested that the presence of the uricase gene mutation was associated with increased serum levels of uric acid, increased antioxidant capacity, and significantly diminished OS, associated with advanced aging and carcinogenesis [38]. Data from studies in various populations, including healthy subjects, smokers, type 1 diabetics, and patients with CVD suggested that infusion of uric acid resulted in significant improvement of total antioxidant capacity and preservation of endothelial function [39,40,41,42,43].

Although there is accumulating evidence highlighting the antioxidant properties of uric acid in the circulation, data from several clinical studies have clearly shown that uric acid is associated with endothelial damage (the first, crucial step towards atherosclerosis), hypertension, and CVD [17,44,45]. While uric acid is a scavenger of oxidants in the extracellular environment, once it gets inside the cell it behaves as a pro-oxidant, via several mechanisms: activation of peroxynitrite-mediated oxidation of lipids [46], stimulation of pro-inflammatory biomarkers, and reduction of the antioxidant nitric oxide (NO) endothelial levels. The mechanisms underlying the paradoxical phenomenon of uric acid inducing OS inside the cell, while acting as an antioxidant in the extracellular environment remain unknown. Uric acid might act as a pro-oxidant in the hydrophobic environment inside the cell, as opposed to the hydrophilic extracellular environment [46]. Another possible explanation might be that after binding of uric acid to pro-oxidants, such as peroxynitrite, the degradation process produces both ROS and alkylating compounds [47] that might easily dissolve into the circulation in the extracellular environment, but in the cellular environment, they could act as free radicals.

After incubation of human aortic cells with soluble uric acid, there was a stimulation of nicotinamide adenine dinucleotide phosphate (NADPH) oxidases, with a subsequent release of free radicals that led to the excessive OS in the mitochondria [10]. Another study showed that after exposure of rat vascular smooth muscle cells to uric acid, there was an excessive accumulation of OS biomarkers, such as 8-isoprostanes and hydrogen peroxides that was partially reversed after incubation with renin-angiotensin-aldosterone system (RAAS) blockers (antihypertensive drugs), such as losartan or captopril [14]. The authors suggested that uric acid triggers OS and vascular damage through the stimulation of the RAAS. In agreement with these results, uric acid triggers excretion of angiotensin II from vascular cells in vitro and stimulates expression of renin in experimental models [7,14,15]. Similarly, Yu et al. found that exposure of human, umbilical vein endothelial cells to uric acid (even at physiological levels of 6 mg/dL) triggered time and dose-dependent oxidative burst-assessed by overproduction of ROS- and subsequent oxidative cell damage leading to apoptosis. Moreover, hyperuricemia-mediated OS activated RAAS to induce further endothelial damage and was successfully abrogated by probenecid, a urate-lowering agent that blocks entry of uric acid into cells [15]. The authors concluded that OS triggered by uric acid in vascular cells might play a role in aging and apoptosis of endothelial cells.

However, uric acid might trigger OS through other molecular pathways, as well, such as renal and systemic inflammation. Inflammation and OS are tightly linked entities, with one causing the other, especially in CKD [48]. Zhou et al. found that induction of hyperuricemia in mice was accompanied by infiltration of the inflammatory macrophages and T-cells in the kidneys and activation of inflammatory biomarkers, such as tumor-necrosis factor alpha (TNF-a), nuclear factor kappa-B (NF-kB), and monocyte chemoattractant protein-1 (MCP-1) [16]. Since it is well established that inflammation triggers the formation of ROS [49], uric acid might induce OS through its pro-inflammatory action. In agreement with these results, another in vitro study showed that the exposure of human endothelial and vascular smooth cells to uric acid was accompanied by upregulation of the inflammation status and the subsequent increase in C-reactive protein (CRP) in the intracellular environment. Moreover, uric acid abrogated the release of the antioxidant NO in human vascular smooth cells. The authors suggested that the entry of uric acid into cells is responsible for the reduction of antioxidant mechanisms and activation of CRP [50].

Data from animal models also showed that hyperuricemic rats developed excessive OS with subsequent damage of renal mitochondrial integrity and the vasculature [10,51]. In another study, normal and nephrectomized rats were treated with oxonic acid (an inducer of hyperuricemia) in order to develop hyperuricemia with or without allopurinol, a uric acid reducer. In normal rats, elevated serum uric acid levels were accompanied by glomerular hypertension, thickening of the afferent renal arteriole, and a 35% decrease in the single nephron glomerular filtration rate, while treatment with allopurinol successfully prevented all morphological and functional changes. Compared to normal, the nephrectomized rats exhibited significantly greater hyperuricemia-induced damage in the renal vasculature. In these animals, treatment with the urate-lowering agent allopurinol could not fully prevent renal vascular injury. The authors of the study suggested that thickening of the renal arteries resulting in glomerular hypertension, ischemia, and tubulointerstitial inflammation and subsequently enhanced OS could be a mechanism explaining the linkage between uric acid, hypertension and CKD [52]. In another study with a similar design, after mild hyperuricemia was induced in normal and 5/6 nephrectomized rats, the authors found that hyperuricemia caused significant changes in systemic and renal hemodynamics, with subsequent loss of renal autoregulation and onset of inflammation and OS in the nephrectomized animals [53]. Similarly, Sanchez-Lozada et al. showed that experimental hyperuricemia in rats triggered the accumulation of free radicals and significantly reduced the synthesis of the antioxidant scavenger NO, as documented by the reduced levels of NO in urine [54]. The authors concluded that even mild hyperuricemia is accompanied by enhanced OS and accelerated endothelial dysfunction.

Moreover, in various settings, several investigators have found that the treatment with XO inhibitors (urate-lowering agents) might also abrogate the development of endothelial dysfunction, a well-known precursor of OS and atheromatosis [55,56,57].

Data from experimental studies suggest that uric acid and hyperuricemia lead to inflammation and OS, activate RAAS, and cause cell apoptosis, hypertension, endothelial, and severe structural injury in the kidney of animals in vivo. Although acting as an antioxidant in the plasma, once it enters the cell, uric acid promotes oxidation of lipids, proteins, DNA, and carbohydrates [47]. Lowering of uric acid can abrogate and partially reverse the OS and uric acid-induced cellular damage.

## 4. The Effect of Antioxidant Supplements in the Reduction of Uric Acid and OS

It is well-established that dietary interventions are crucial for lowering serum uric acid. Similarly, diets rich in antioxidants could reduce OS. Following this path, several investigators proposed supplementation with exogenous antioxidants to counteract the deleterious synergistic effects of hyperuricemia and OS.

## 5. Vitamins C and E

Vitamin E is a fat-soluble vitamin containing eight compounds, four tocopherols and four tocotrienols with pleiotropic health benefits and strong antioxidant capacity. Vitamin C (or ascorbic acid) is an essential nutrient with scavenging activity. It binds directly to the superoxide anion and hydroxyl radicals and de-activates them. It is known from epidemiological studies that one of the first lifestyle changes suggested for hyperuricemic patients is increased dietary intake of fruits and vegetables that are rich in Vitamin C [58]. Vitamin C, uric acid, and OS are involved in a complex interrelationship. Every time uric acid prevents oxidative neutralization of the antioxidant SOD, a urate radical is formed. This compound does not bind with oxygen like other free radicals [37] and is regenerated to urate by ascorbic acid [59]. Therefore, ascorbic acid has been suggested to enhance the antioxidant effects of uric acid [59,60] and serum urate has been shown to stabilize ascorbic acid in the tissues [61]. Data from in vitro and in vivo studies suggest that Vitamin C reduces serum uric acid levels through several mechanisms by inhibiting uric acid synthesis [62], by increasing urinary excretion [63] and fractional renal clearance of uric acid [64], and by directly decreasing ROS-derived cell damage [65,66]. Moreover, Vitamin C is able to regenerate the active form of the antioxidant Vitamin E and therefore combined therapy with both vitamins results in a synergistic antioxidant, anti-inflammatory and anti-atherogenic effect [67]. Vitamin E is a lipid-soluble nutrient that protects the integrity of cell membranes by scavenging ROS and blocking the chain of oxidative reactions leading to OS injury of cells. It has been shown in diabetics that after a standardized meal, there is an overproduction of free radicals -assessed by malondialdehyde (MDA)- in plasma, accompanied by a reduction in Vitamin E levels. Therefore, dietary supplementation of Vitamin E could ameliorate meal-generated OS in these high-risk patients [68].

### 5.1. Combination of Vitamin C and E

In vivo, after rats were subjected to ethanol (a pro-oxidant chemical compound also known as ethyl alcohol)-derived OS, uric acid serum levels were elevated, and local and systemic OS status significantly enhanced. However, these effects of ethanol were successfully reversed after three days of supplementation with a combination of antioxidants (ascorbic acid, a-tocopherol and, selenium). The authors found that the intake of these antioxidants improved the ethanol OS-induced hyperuricemia, probably through their antioxidant activity [69]. Similarly, Rodrigues et al. found that hypoxanthine (a key enzyme responsible for the generation of uric acid in the metabolism of purines) triggered an acute oxidative burst in the renal cortex and medulla of rats, by reducing the antioxidant defense mechanisms and promoting lipid peroxidation. Administration of the urate-lowering agent allopurinol alone or together with the antioxidant Vitamins C + E abrogated most of the OS alterations observed [70]. Moreover, allopurinol seemed to improve OS status to a greater extent compared to exogenous antioxidant vitamins. This was probably due to the fact that allopurinol abrogates the production of these superoxide anions by directly blocking XO, while the antioxidant Vitamins C + E merely try to sweep up the excessive ROS that has been formed and released to the circulation. Since both allopurinol and the antioxidant vitamins ameliorated the oxidative damage elicited by hypoxanthine, via different mechanisms, the authors proposed supplementation with antioxidants in addition to standard therapy with allopurinol for the treatment of hyperuricemic OS. On the other hand, data from the Nutrition and Health Survey in Taiwan (NAHSIT), a large cohort, a nationwide survey on 2176 adults from the general population showed that there was no association between dietary intake of Vitamin C and E and serum levels of uric acid [71]. This lack of association could be attributed to several reasons, such as the observational design of the study and the fact that the plasma levels of Vitamins C + E were not determined. Furthermore, the dietary intake of the vitamins was evaluated by food questionnaires and 24-h diet recall, and therefore, the data collected might not be precise.

### 5.2. Vitamin C

Epidemiological data from a cross-sectional study in 9010 subjects from the general population showed that the dietary intake of Vitamin C was significantly lower in hyperuricemic subjects, compared to those with normal serum uric acid [72]. Another large, prospective study proposed a close relationship between high dietary Vitamin C intake and low future risk of gout in men. The increased dietary Vitamin C intake of 1.5 g per day conferred a 45% lower risk of incidence of gout [73]. Several investigators have suggested that Vitamin C has uricosuric properties, and therefore, might be considered for the management of hyperuricemia. Stein et al. first showed that an increased dose of ascorbic acid (4 g orally) doubled the urinary excretion of uric acid in 14 healthy volunteers [64]. Similarly, large doses of 3 g per os daily intake of ascorbic acid in six healthy subjects for a week resulted in a transient increase in urinary removal of uric acid [74]. Since ascorbic and uric acid are both reabsorbed by the kidney in the proximal tubule, it is hypothesized that ascorbic acid intake induces increased urine excretion and decreased renal reabsorption of uric acid due to the competition by ascorbic acid for the renal transport at a common proximal tubular site [75]. 

Huang et al. conducted a randomized, double-blind, placebo-controlled study to investigate the possible impact of Vitamin C intake on serum uric acid levels. One-hundred and eighty-four healthy volunteers were randomized to receive either placebo or Vitamin C (500 mg orally daily) for two months. At the end of the study period, supplementation with Vitamin C decreased serum uric acid levels and increased eGFR [76]. Similarly, two observational general population-based studies in 1387 and 68 men, respectively, showed a significant inverse association between serum uric acid levels and plasma ascorbic concentration [77] or dietary intake of Vitamin C [65]. To assess the effect of Vitamin C supplementation on serum acid concentrations in dialysis patients, Biniaz et al. performed a randomized, multicenter, placebo-controlled study on 172 patients undergoing maintenance HD. At baseline, nearly half (46.75%) of the patients included in the study had uric acid serum levels above 6 mg/dL. All patients were randomly allocated to the treatment group that received intravenous 250 mg of Vitamin C thrice weekly after every HD session for two months and the two control groups that received either nothing or placebo intravenous normal saline. After the study period, compared to controls, the Vitamin C group exhibited a significant decrease in serum uric acid concentrations [78]. Moreover, Vitamin C supplementation resulted in a significant decrease of circulating CRP levels [79]. The authors concluded that since HD is a state of enhanced OS and inflammation characterized by decreased antioxidant activity and high incidence of hyperuricemia, Vitamin C supplementation could have multiple beneficial effects in chronic HD patients. Another randomized study found that compared to placebo, daily intravenous administration of Vitamin C (500 mg for 10 days) in patients with acute ischemic stroke resulted in a significant reduction of OS biomarkers and uric acid serum levels [80]. In disagreement with these results, another randomized controlled trial enrolled 40 patients with gout and serum uric acid above 6 mg/dL and found that compared to allopurinol, Vitamin C (500 mg/day for eight weeks) supplementation had no significant effect on serum levels of uric acid and uric acid urinary excretion [81]. These findings could be explained due to several factors: firstly, the uricosuric effect of Vitamin C could be weak, secondly the dose of Vitamin C was relatively small, and thirdly, several of the study participants received other medications, such as diuretics and acetylsalicylic acid that could interact with Vitamin C. To investigate the effect or oral Vitamin C administration on serum levels of uric acid, Juraschek et al. performed a meta-analysis that included 13 randomized controlled trials on 556 subjects [63] and concluded that Vitamin C supplementation was accompanied by a significant decrease in serum uric acid levels. Moreover, trials that administered Vitamin C at a dose of 500 mg/day or higher showed a more pronounced effect on serum uric acid levels.

### 5.3. Vitamin E

Animal models of experimental hypertension exhibited hyperuricemia and a decrease in urinary excretion of uric acid within a week. However, treatment with Vitamin E lowered blood pressure, decreased uric acid in the serum, and had a significant uricosuric effect [82]. The combined treatment of Vitamin E and allopurinol successfully abrogated the oxidative response that was triggered in rats’ liver and brain after exposure to cypermethrin (a broad-spectrum insecticide, highly toxic to human and animals) [83].

Kuroda et al. failed to show any association between serum uric acid and vitamin E levels (well-known antioxidants) and the total antioxidant capacity in a cohort of 111 patients with CKD (13 in stage 1–4, 11 ESRD and 87 HD) [84]. Since OS is progressively enhanced in CKD and further exacerbated in dialysis patients, several investigators explored whether supplementation with Vitamin E could have antioxidant and hypouricemic effect in dialysis patients. In a recent systematic review and meta-analysis, D’Arrigo et al. investigated the effects of Vitamin E coated versus conventional membranes in HD patients. Sixty studies and 2118 patients were included. Although the use of Vitamin E membranes was found to decrease inflammation and OS significantly, it had no impact on serum uric acid levels [85]. However, in a small cohort of 14 PD patients, daily oral intake of 400 mg of Vitamin E reduced the plasma MDA levels but failed to alter the uric acid serum concentration [86]. This could be attributed to the fact that serum levels of uric acid were within the normal range during the whole study.

Vitamin C is the most studied dietary antioxidant. Through several mechanisms, Vitamin C can abrogate OS and reduce serum uric acid levels. There is a growing body of evidence suggesting that daily supplementation of Vitamin C combined with a standard urate-lowering therapy could be beneficial for hyperuricemic patients.

## 6. Polyphenols

Plant-derived polyphenols are compounds offering protection against aging, cancer, atherosclerosis, and diabetes, probably due to their antioxidant and anti-inflammatory properties [87]. Cicero et al. conducted a randomized, double-blind, placebo-controlled clinical trial to investigate the effect of apple polyphenols on uric acid, compared with the standard urate-lowering agent, allopurinol. Sixty-two overweight subjects were randomly allocated to either receiving 300 mg of apple polyphenols daily via oral intake or placebo for two months. Treatment with polyphenols significantly reduced circulating uric acid and had a protective effect against vascular OS, probably through inhibition of the enzyme XO [88]. There is accumulating data suggesting that bergamot derived polyphenols, could be used for management of hyperuricemia and amelioration of uric acid-derived OS, through their dual effect as XO inhibitors and antioxidants as ROS scavengers [89].

## 7. Flavonoids

Flavonoids are natural substances included in polyphenolic structures, found in vegetables, fruits, tea, grains, and wine. They are the most studied group of polyphenols. Besides their anti-inflammatory and anti-oxidative properties, flavonoids are involved in the activity of important enzymes, such as XO. Due to their molecular structure, these natural antioxidants act as direct superoxide scavengers and XO inhibitors, resulting in the suppression of ROS and uric acid formation [90,91].

There is a growing body of evidence showing that flavonoids exert significant antioxidant and hypouricemic activities in vitro and in vivo. In vitro, the flavonoids luteolin, silibinin and quercetin were found to be competitive inhibitors of bovine XO. Moreover, quercetin and luteolin decrease the rate of superoxide anion formation by XO and lower uric acid levels [92]. In vitro, supraphysiological doses of quercetin were also found to be 100 times more powerful in scavenging ROS and inhibiting XO activity compared to allopurinol (a uric acid reducer) [93]. In vivo, it has been shown that flavonoids can reduce uric acid levels, through inactivation or downregulation of the enzyme XO [94,95]. Moreover, the inhibitory activity of flavonoids on XO is stronger than their direct scavenging ability of free radicals [96]. However, Huang et al. suggested that flavonoid-including quercetin might not affect uric acid levels in normal and hyperuricemic mice [97]. Of all 15 flavonoids, quercetin was able to exert urate-lowering properties even at very low doses, in experimental models [95]. Similarly, oral administration of quercetin to chemically-induced hyperuricemic mice was accompanied by a dose-dependent hypouricemic effect, through inactivation of xanthine dehydrogenase/XO activities [98]. Moreover, in mice subjected to chemically induced hyperuricemia, administration of quercetin lowered uric acid levels by increasing its urine excretion and protected the kidney from hyperuricemic damage. The authors suggested another molecular pathway underlying the uricosuric and nephroprotective functions of quercetin, through regulation of organic ion transporters and uromodulin expression [99]. A recent study showed that supplementation of the flavonoid-rich fraction of a Chinese herb, Smilax glabra Roxb, in Sprague Dawley rats with uric acid nephropathy for five weeks resulted in either reduction of uric acid generation or enhanced urinary excretion, by activating organic anion transporters [100]. Similarly, quercetin exhibited increased urinary removal, decreased serum urate levels, and upregulation of organic anion renal transporters, in hyperuricemic rodents. The results of this study highlighted that this dietary flavonoid might be beneficial for the management of hyperuricemia with kidney damage [101]. Moreover, in another similar animal study, the treatment with this flavonoid supplement significantly reversed urate-induced renal injury and inhibited renal and systemic OS and inflammation status. The authors concluded that due to their effect on urate excretion through the urine, flavonoids could suppress renal OS, inflammation and damage in uric acid nephropathy rats [100]. Haidari et al. randomized 30 normal and 30 hyperuricemic Wistar rats to receive standard diet (controls), allopurinol, or quercetin once daily per os for two weeks. Although quercetin had no effect on the uric acid of normal rats, it significantly decreased serum uric acid levels of the hyperuricemic animals in a time-dependent pattern, through the inactivation of XO. Moreover, the treatment with flavonoids was accompanied by a significant rise in total antioxidant capacity and reduction of malondialdehyde levels of hyperuricemic rats. Although the urate-lowering effect of allopurinol was much stronger than that of quercetin, it had no beneficial effect on OS biomarkers [102]. In animal models with induced diabetic nephropathy and hyperuricemia, quercetin and allopurinol were found to ameliorate OS, prevent kidney damage and suppress renal inflammation, via their hyperuricemic effect [103]. In another study, Renugadevi et al. induced cadmium (a divalent metal highly toxic to humans and animals) nephrotoxicity in rats. Uric acid levels were significantly elevated in serum and decreased in urine. Cadmium-mediated OS in the kidney and circulation was documented by a significant and acute increase of pro-oxidative biomarkers and a decrease of enzymic antioxidants. The kidneys of the rats that received cadmium exhibited severe tubulointerstitial necrosis and glomerular damage. Quercetin treatment prevented renal structural and functional damage caused by cadmium, restored uric acid levels to normal and suppressed OS status. The authors concluded that this flavonoid, through its antioxidant properties, could decrease the OS-induced elevated uric acid in the serum of rats [104]. Shi et al. conducted a randomized, double-blind, placebo-controlled, cross-over trial to investigate the effect of quercetin on uric acid. Twenty-two pre-hyperuricemic patients (i.e., with uric acid in the high normal levels) were randomly divided to 500 mg of daily oral quercetin or placebo for four weeks, with a wash-out period of one month between treatments and the change in serum uric acid was assessed [105]. The authors found that treatment with quercetin significantly lowered plasma uric acid levels by −26.5 μmol/L (95% CI, −7.6 to −45.5, p = 0.008).

Dietary flavonoids reduced uric acid levels, suppressed OS, and protected from kidney damage in multiple animal studies. Since allopurinol failed to exert antioxidant properties, it is interesting to hypothesize that combination therapy of allopurinol and selected flavonoids (such as quercetin) might have pleiotropic effects on hyperuricemic CKD patients. 

## 8. Tea

Tea, the leaf of *Camelia sinensis*, remains the most widely consumed drink worldwide, excluding water. Of the six types of processed tea, consumption of black and green tea has been shown to exert certain beneficial health effects. The main chemical components of tea are flavonoids (phenolic compounds, mainly catechins), well known ROS scavengers. After tea consumption, tea catechins cause a significant increase in antioxidant capacity in both plasma and gut [106]. Moreover, five catechins and two flavonoid compounds that are found in tea can successfully inhibit the activity of the enzyme XO [107]. Data from animal studies have shown that in hyperuricemic mice, green tea polyphenols could decrease the serum concentration of uric acid in a dose-dependent manner, through the inhibition of XO liver expression and modification of renal urate-anion transporters [108]. Since XO is involved in purine metabolism and generates ROS and uric acid, it is suggested that besides antioxidant, tea might also exert hypouricemic properties.

However, epidemiological data from the National Health and Nutrition Examination Survey (NHANES) study in 14,758 general population subjects showed that only coffee and not tea consumption was associated with decreased serum uric acid levels and a lower incidence of hyperuricemia [109]. In agreement with these results, a Korean epidemiologic study in 9400 general population participants showed no effect of coffee or tea consumption on the risk of hyperuricemia [110]. Similarly, a cross-sectional, multicenter study in 2240 middle-aged adults showed that only coffee and not green tea consumption was inversely related to decreased serum acid levels [111]. In disagreement with these results, in 483 general population subjects, Teng et al. found that compared to non-drinkers, those who consumed green tea daily presented a dose-dependent and two-fold increase with hyperuricemia [112]. However, black tea or coffee consumption failed to show any association with hyperuricemia. To investigate the possible hypouricemic and antioxidant effects of green tea in 30 healthy volunteers, Jatuworapruk et al. conducted a randomized study with seven days wash-out, 14 days intervention, and seven days follow-up design. The authors found that green tea caused a modest decrease in both serum uric acid levels and uric acid clearance. Moreover, green tea significantly increased circulating antioxidant capacity in a dose-dependent manner [113]. Furthermore, black tea intake was found to cause a significant reduction in uric acid serum levels and serum CRP levels in patients susceptible to cardiovascular disease [114]. However, a systematic review and meta-analysis of 15 observational studies reported that green tea intake was not related to the incidence of gout and hyperuricemia or serum uric acid levels [115].

The catechins in black and green tea scavenge free radicals and exert multiple beneficial health effects. Although in vitro and in vivo studies showed that tea seemed to inhibit the activity of XO, and therefore, decrease serum uric acid levels, the hypouricemic effect of tea was not established in human trials, and therefore, no recommendation could be established.

## 9. Curcumin

Curcumin is a natural polyphenol, extracted from the herbal spice turmeric. During the past decade, it has gained scientific interest due to its beneficial antioxidative and anti-inflammatory properties. In CKD and HD patients, curcumin has been shown to act as a scavenger of free radicals, resulting in a significant suppression of OS [5]. Since XO (the enzyme responsible for the formation of uric acid) is involved in generating ROS, it has been hypothesized that administration of the antioxidant curcumin could have a urate-lowering effect. In vitro, the local administration of curcumin significantly and directly inhibited XO activity, and therefore, caused a subsequent reduction in uric acid levels [116,117]. More recent studies proposed that not only curcumin binds directly to XO, but also its degradation by-products exhibit important inhibitory properties against XO [117]. However, another study failed to show any effect of curcumin on purified bovine XO activity [92]. Data from in vivo studies regarding the urate-lowering effect of curcumin are limited. Al-Rubaei et al. randomly assigned rats to three groups. Group A was the control group. Group B was subjected to chemically induced OS for 60 days. Group C also experienced OS for two months, followed by daily treatment with curcumin for another 30 days. Compared to controls, group B rats had significantly increased levels of pro-oxidative biomarkers (such as MDA, 8-hydroxy-2-deoxyguanosine), decreased concentrations of the antioxidants superoxide dismutase, catalase, and reduced glutathione. Moreover, compared to group A, group B animals had significantly increased levels of uric acid and the pro-inflammatory marker, TNF-a. Treatment with curcumin successfully improved all OS and inflammation parameters and lowered uric acid to nearly normal levels. The authors concluded that the urate-lowering effect of curcumin could be due to its strong antioxidant capacity [118]. In a randomized, controlled study, 100 patients with non-alcoholic fatty liver disease (NAFLD)—a condition characterized by an enhanced OS and inflammation state—were randomly allocated to either receive curcumin (500 mg twice daily) or nothing for two months. Compared to controls, the group that was treated with curcumin exhibited significantly lower serum levels of uric acid. Even after adjustment for several co-founders, treatment with curcumin remained a significant strong predictive factor of uric acid reduction [119].

Curcumin, a natural antioxidant might have a urate-lowering effect, through direct binding to XO or by suppressing inflammation and OS. The data regarding the effect of curcumin on uric acid levels are very limited but seem quite promising.

## 10. Probiotics

Probiotics are yeasts and live bacteria that are thought to exert various beneficial health effects. During the past decade, there is a growing body of evidence suggesting that the disruption of natural bacteria balance in the gastrointestinal system (known as the gut microbiome) could lead to multiple adverse events. Dietary supplements containing probiotics are thought to restore the gut microbiome balance when it is disrupted by conditions such as CKD. In renal impairment, the excretion of uric acid is directly associated with gut uricolysis by the gut microbiome. A recent randomized controlled animal study aimed to investigate the effect of probiotic supplements on uric acid levels and uric acid-derived kidney injury. Thirty rats were randomly divided to the following five groups: control, induced hyperuricemia and standard diet, induced hyperuricemia and placebo, and induced hyperuricemia treated with two different probiotic formulas containing uricolytic bacteria. After five weeks of follow-up, the authors found that chemically-induced hyperuricemia caused hypertension, functional, and morphological changes in the kidney, and alterations in fecal microbiota. Probiotic supplements successfully inhibited hyperuricemia, prevented the accumulation of uric acid in the kidneys, decreased uric acid urine excretion, and prevented systemic and renal cortex OS induced by hyperuricemia. Moreover, the inhibitory effect of probiotics on OS and uric acid was accompanied by the prevention of the renal damage and hypertension caused by hyperuricemia [120]. Two prospective, randomized, double-blind, placebo-controlled crossover trials investigated the effect of probiotic supplements—that included bacteria selected to metabolize uric acid and nitrogenous wastes—to CKD progression and hyperuricemia in patients with renal impairment. The first was a single-center trial and showed that six months of daily, oral supplementation with probiotics in 16 patients with CKD stage 3 and 4, successfully reduced urea and uric acid serum levels [121]. The second study was carried out in multiple centers across four countries, included 46 patients with CKD stages 3 and 4, and showed that six months treatment with probiotics (daily, orally) significantly decreased serum urea levels in 63% of the patients and reduced uric acid levels in 33% of them; however, without statistical significance [122]. In disagreement with these results, Asemi et al. performed a randomized, double-blind, placebo-controlled study on 54 diabetics and found that although the intake of probiotic supplements for two months was accompanied with a significant decrease of OS and inflammatory biomarkers, the levels of uric acid remained unchanged [123]. Similarly, another randomized, double-blind, placebo-controlled, cross-over trial in HD patients showed that a two-month treatment with probiotics was safe, well-tolerated, reduced pro-inflammatory markers but failed to decrease uric acid serum levels [124].

In CKD settings, probiotic supplements containing uricolytic bacteria have been associated with reduced OS. However, the data on the effect of probiotics on uric acid levels remains controversial. Since probiotic supplements are thought to be well-tolerated and safe in these patients, further trials in large cohorts are needed in order to elucidate this relationship.

## 11. l-Arginine

NO, through its antioxidant properties, protects renal function by preserving tubuloglomerular balance, raising renal blood flow, and ensuring homeostasis of electrolytes and fluids. NO availability in the endothelium defines a healthy and well-functioning vasculature. Free radicals lead to the inactivation of NO, and therefore, affect its availability directly, causing vascular damage. ΝO deficiency is a well-established promoter of OS and has been repeatedly associated with progression of CKD and onset of endothelial dysfunction in uremic patients [4]. L-Arginine is an amino acid with antioxidant properties, scavenging ROS directly, and acting as a substrate for ΝO synthesis. Several investigators showed both, in uremic animal models and humans that supplementation of l-arginine successfully reduces OS status and inhibits vascular calcification, through upregulation of NO [125,126]. Sanchez-Lozada et al. investigated the possible effect of chronic and acute supplementation of l-arginine on the structural and functional changes in renal hemodynamics that was caused by induced hyperuricemia in rats [54]. To induce hyperuricemia, oxonic acid was administered in thirty-one rats, once daily, and they were followed for five weeks. All hyperuricemic rats were divided into four groups: the control group that did not receive any supplement (*n* = 8), those who were infused acutely, once with 15 mg/kg/min l-arginine (*n* = 9) and the chronic treatment groups that received l-arginine at low (1%) and high (2.5%) dosages for five weeks. Acute infusion of l-arginine reversed the injury of the renal arteries and caused vasodilation with a subsequent rise in single nephron GFR. However, glomerular hypertension was not abrogated, and therefore, the autoregulation of the kidney was significantly impaired. Chronic supplementation of l-arginine, especially at the high dose, successfully prevented all glomerular and systemic alterations induced by hyperuricemia. The beneficial effects of l-arginine were attributed to the stimulation of NO production that was documented by increased NO in urine.

## 12. N-Acetylcysteine (NAC)

NAC is a powerful scavenger of ROS that protects cells and tissues from oxidative damage and has repeatedly been shown to be an effective antioxidant in CKD patients [5]. Data from in vitro and in vivo trials suggest that NAC might also prevent the OS caused by hyperuricemia. In human vascular endothelial cells, uric acid stimulated overproduction of ROS, intracellular OS, and subsequent cell death. Treatment with the antioxidant NAC (even at a small dose of 10 mmol/L) successfully ameliorated the apoptotic procedure mediated by hyperuricemia and oxidative stress [15]. Similarly, hyperuricemic mice exhibited enhanced OS and increased insulin resistance. Administration of NAC directly blocked the hyperuricemic-induced insulin resistance and suppressed both OS and uric acid levels [127].

## 13. Conclusions

Although acting in plasma as an antioxidant, once it enters the intracellular environment, uric acid behaves as a pro-oxidant. Uric acid is tightly linked with the onset and development of OS, especially in subjects susceptible to oxidative damage, such as CKD patients. Besides standard uric-acid lowering therapy, several antioxidants have been proposed to reduce both OS and uric acid serum levels further. The data regarding the effects of antioxidants on uric acid and OS biomarkers are limited and mainly derived from animal studies or small observational trials. The daily intake of curcumin, l-arginine, and Vitamin C, in combination with standard therapy, might act synergistically and ameliorate the deleterious effects of OS and hyperuricemia. Further, large, prospective, randomized controlled studies are needed in order to draw definite conclusions.

## Figures and Tables

**Table 1:** The effect of elevated serum uric acid in the kidney.

Pathogenetic Mechanisms	Effects on Kidney	Systemic Effects
Accumulation of uric crystals [7]	Glomerular hypertension	Hypertension
Stimulation of RAAS [14,15]	Glomerular hypertrophy-sclerosis	Diabetes mellitus
Stimulation of T-cells and macrophages [7,16]	Tubulointerstitial disease	Obesity
Activation of MCP-1, NkF-kB, TNF-a [16]	Renal arteriolar sclerosis-ischemia	Non-alcoholic fatty liver disease
Activation of NO, NADPH oxidase [10]	Renal ischemia	Inflammation
Mitochondrial dysfunction [10]	Albuminuria	Metabolic syndrome
Phenotypic change of renal tubular cells [8,9,11]	Loss of eGFR	Arteriosclerosis
Endothelial dysfunction [10,13,15,17]	Acute kidney disease	Oxidative stress
